# Performance of the renal resistive index and usual clinical indicators in predicting persistent AKI

**DOI:** 10.1080/0886022X.2022.2147437

**Published:** 2022-11-16

**Authors:** You Fu, Cong He, Lijing Jia, Chen Ge, Ling Long, Yinxiang Bai, Na Zhang, Quansheng Du, Limin Shen, Heling Zhao

**Affiliations:** a Department of Critical Care Medicine, Hebei Medical University, Shijiazhuang City, China; b Department of Intensive Care Unit, Hebei General Hospital, Shijiazhuang City, China

**Keywords:** Acute kidney injury, renal resistive index, kidney prognosis, septic shock, critical care, ultrasonography

## Abstract

**Background:**

Early recognition of persistent acute kidney injury (AKI) could optimize management and prevent deterioration of kidney function. The Doppler-based renal resistive index (RI) has shown promising results for predicting persistent AKI in preliminary studies. Here, we aimed to evaluate the performance of renal RI, clinical indicators, and their combinations to predict short-term kidney prognosis in septic shock patients.

**Method:**

We performed a retrospective study based on data from a prospective study in a single-center general ICU between November 2017 and October 2018. Patients with septic shock were included. Clinical indicators were evaluated immediately at inclusion, and renal RI was measured within the first 12 h of ICU admission after hemodynamic stabilization. Persistent AKI was defined as AKI without recovery within 72 h. A multivariable logistic regression was used to select significant variables associated with persistent AKI. The discriminative power was evaluated by a receiver operating characteristic curve analysis.

**Result:**

Overall, 102 patients were included, 39 of whom had persistent AKI. Renal RI was higher in the persistent AKI patients than in those without persistent AKI: 0.70 ± 0.05 vs. 0.66 ± 0.05; *p* = 0.001. The performance of RI to predict persistent AKI was poor, with an area under the receiver operating characteristic curve (AUROC) of 0.699 [95% confidence interval (CI) 0.600–0.786]. A clinical prediction model combining serum creatinine at inclusion and the nonrenal SOFA score showed a better prediction ability for nonrecovery, with an AUROC of 0.877 (95% CI 0.797–0.933, *p* = 0.0012). The addition of renal RI to this model did not improve the predictive performance.

**Conclusion:**

The Doppler-based renal resistive index performed poorly in predicting persistent AKI and did not improve the clinical prediction provided by a combination of serum creatinine at inclusion and the nonrenal SOFA score in patients with septic shock.

## Introduction

Acute kidney injury (AKI) is a common and severe complication in critically ill patients, especially in patients with sepsis, who account for up to 50% of all patients [1,2]. The development of AKI is associated with increased morbidity and short-term mortality [3]. Early recognition of AKI without short-term recovery is challenging but crucial in ICU clinical practice, as the distinction between transient and persistent AKI might help to optimize treatment, such as promptly restoring kidney perfusion, limiting fluids, and avoiding nephrotoxic agents and might help with patients facing kidney replacement therapy (KRT) [4], which then can improve the outcome [5,6].

Usual markers of kidney dysfunction, namely, insensitive serum creatinine (sCr) or nonspecific oliguria, limit the early diagnosis, differential diagnosis, or prognostic evaluation of AKI [7]. Some urinary indexes and biomarkers, such as fractional excretion of urea, tissue inhibitor of metalloproteinases-2 and insulin-like growth factor-binding protein 7 ([TIMP-2]*[IGFBP7]), have been assessed for the early prediction of persistent AKI. However, these indicators perform poorly and cannot be recommended for widespread use in clinical practice [8–10].

Vascular dysfunction may play a vital role in the pathophysiological mechanisms of septic AKI [11]. Renal Doppler ultrasound can measure the renal resistive index (RI), which is a sonographic index that reflects alterations in the blood flow profile of the intrarenal arcuate or interlobar arteries, allowing us to explore kidney hemodynamics noninvasively. RI has been used to assess kidney perfusion [12,13] and to predict kidney dysfunction [14–16] and recovery from AKI in the ICU [16,17]. Several studies have shown its efficiency in predicting persistent AKI [15,16,18]; however, large heterogeneity between these studies was noted [17]. In addition, AKI is a complex condition, and it is unlikely that any single marker of kidney injury will be able to fully predict the changes in kidney function. Some clinical variables, such as age or comorbid diabetes, contributing to hardening of the arteries have been associated with AKI and its short-term prognosis [19]. Similarly, fluid accumulation has been demonstrated to be a negative predictor for the recovery of kidney function because it may cause hypoxemic injury to the kidney [20]. Furthermore, as a systemic perfusion indicator, lactate, can reflect kidney hypoperfusion to some extent [21], which may have an impact on the reversibility of AKI. Some studies have shown that the severity of illness may also help to predict kidney recovery from AKI [1,22]. The value of combining renal RI with clinical indicators has not been studied in the early prediction of persistent AKI. Therefore, the main objective of this study was to evaluate the ability of renal RI, clinical indicators, and especially the combination of these parameters to predict persistent AKI in patients with septic shock.

## Methods

### Data source and study population

For a retrospective study, we used the data of the patients with septic shock from a previous prospective study, which was conducted to evaluate the ability of renal RI and central venous pressure (CVP) to predict AKI in a single-center general ICU between November 2017 and October 2018 [23]. The study protocol was approved by Hebei General Hospital Institutional Review Board (No. 2017–126), which waived the need for signed informed consent, given the noninterventional study design. In that previous study, all consecutive patients with septic shock according to the Third International Consensus Criteria [24] were included immediately after admission to the ICU in Hebei General Hospital. The exclusion criteria were age <18 years, pregnancy, hospital stay <24 h, having already recovered from AKI according to our definition, chronic kidney disease stage IV–V, history of kidney transplantation, and no central venous catheterization of the internal jugular vein or subclavian vein. Patients with confirmed renal artery stenosis or renal vein thrombosis and cardiac arrhythmia were also excluded because these conditions might preclude renal Doppler measurement. The primary end-point of the previous study was the onset of AKI. Although the study was open, the investigators were blinded to the primary objective of this study, and patient management was at the discretion of the physician in charge, who was blinded to the renal RI.

For the current study, all the patients who were enrolled in the previous study were included. This study excluded the patients who in imminent need of KRT after ICU admission and those in whom the nature of AKI recovery was nonevaluable at 72 h after inclusion (i.e. no available sCr could be used to classify AKI because of discharge from ICU).

### Definitions

Sepsis was defined according to the Third International Consensus Criteria as the presence of an infection (with a likelihood of at least possible) and organ dysfunction(s) represented by two or more Sequential Organ Failure Assessment (SOFA) points. Patients with septic shock could be identified with a clinical construct of sepsis with persisting hypotension requiring vasopressors to maintain a mean arterial pressure (MAP) of ≥65 mmHg and having a serum lactate level >2 mmol/L (18 mg/dL) despite adequate volume resuscitation [24].

AKI was defined at study inclusion according to the Kidney Disease Improving Global Outcomes (KDIGO) classification using both the creatinine and urine output criteria. AKI was diagnosed by either an increase in sCr greater than or equal to 26.4 µmol/L or an sCr greater than 1.5 times from baseline or a urine output less than 0.5 mL/kg/h for 6 h. AKI severity was determined separately and was classified as stage 1, stage 2, or stage 3 based on the changes in the creatinine concentration or urine output according to the KDIGO guidelines [25]. For each patient, baseline sCr was defined according to the sCr measured the year prior to admission to the ICU. When the baseline sCr level was unknown, this variable was estimated according to the Modification of Diet Renal Disease (MDRD) formula back-calculation [26].

Transient AKI was defined as AKI with recovery occurring within 72 h after inclusion [5,16]. Recovery from AKI was defined as a decrease of at least one stage in AKI severity according to the KDIGO criteria (i.e. decrease of the sCr, reversal of oliguria in the absence of diuretic therapy, and absence of KRT). Persistent AKI was defined as persistent oliguria and/or as a steady or a higher AKI KDIGO stage [16]. When no available sCr could be used to classify AKI because of death, AKI was classified as persistent if the patient met one of the KDIGO criteria for their last measurement. The duration of AKI was measured from its occurrence, which was generally considered to be the time that it was first diagnosed. For the patients who had been diagnosed with AKI at ICU admission, the duration of AKI was calculated from their ICU admission.

Hemodynamic stabilization was considered when the MAP was at least at 65 mmHg without the need for a bolus of fluids and/or the initiation of a vasopressor or an increase in the patient’s vasopressor dose within the last 6 h [15,16].

Diuretic use was defined as the use of diuretics at any time during the first 24 h in the ICU.

### Study protocol and data collection

All eligible patients were treated according to the 2016 Surviving Sepsis campaign guidelines [27]. Serum creatinine was measured at inclusion and then at least once daily, and urine output was recorded hourly for patients with an indwelling urinary catheter based on our standard procedures. Assessments of kidney function were performed at study inclusion and during the next 72 h according to the serum creatinine and/or urine output. AKI was only determined by creatinine elevation in patients who did not have their hourly urine output measured *via* an unlocated urinary catheter.

The patients were divided into persistent AKI (P-AKI) and nonpersistent AKI (no P-AKI) groups based on our definition for persistent AKI. Because no difference was observed between the renal RI values in the patients without AKI and those in the patients with transient AKI in a previous study [16], these patients were assigned to one group, namely, the no P-AKI group.

Demographic data, such as age and sex, were recorded, together with any history of hypertension, diabetes, or chronic kidney disease. The baseline serum creatinine and its means of estimation, mode for ICU admission, time from vasopressor initiation to inclusion, and sepsis sources were collected. The following clinical indicators measured at inclusion were also noted: heart rate, MAP, sCr, hemoglobin, platelet count, total bilirubin, ratio of arterial oxygen partial pressure to fractional inspired oxygen, partial pressure of carbon dioxide, lactate, procalcitonin, CVP, norepinephrine dose and the need for mechanical ventilation. During patient management, the time from inclusion to hemodynamic stabilization, the amount of fluid therapy received within the first 24 h, and the use of diuretics were collected. Patients who were given nephrotoxic agents, including angiotensin II converting enzyme inhibitors or angiotensin receptor blockers, iodinated contrast agents, aminoglycosides, and glycopeptides, were also recorded. The severity of illness was determined using the Acute Physiology and Chronic Health Evaluation (APACHE II) and SOFA score at ICU admission. The SOFA score without a renal component was calculated simultaneously. Two investigators blinded to the primary objective of this study were responsible for collecting the data.

In each patient, renal sonography for RI measurement was performed within the first 12 h of ICU admission and after hemodynamic stabilization. The RI was calculated using a 2–5 MHz curved array transducer (Compact Xtreme CX50, Philips Medical Systems, Bothell, WA, USA) by two investigators (all certified physicians who had received training on the Doppler evaluation of kidney perfusion from the Chinese Critical Ultrasound Study Group). The B mode allowed kidney localization and the detection of signs of chronic kidney disease. A longitudinal view of the kidney was obtained in B-mode US in the posterolateral approach, and color Doppler allowed for vessel localization. An interlobar or arcuate artery was identified and then selected. Pulse-wave Doppler was used to measure blood velocities. The Doppler spectrum was considered optimal when at least three similar consecutive waveforms were visualized. The peak systolic velocity (Vmax) and the minimal diastolic velocity (Vmin) were recorded, and the RI was calculated with the following formula: (Vmax–Vmin)/Vmax. Three measurements were averaged to obtain the mean RI values used for the study[13]. The right kidney was generally chosen for measurement. If the RI of the right kidney was not obtained, the left side could be selected.

### Statistical analysis

Continuous variables were described as the mean ± standard deviation (SD) or median and interquartile range (IQR) based on their distribution. Categorical variables were expressed as frequencies and proportions. Comparisons between groups were assessed by unpaired *t test* or Mann–Whitney test. Categorical variables were analyzed using the chi-square test or Fisher’s exact test.

Based on previous findings [16,28], the sample size was calculated as follows. Assuming an RRI of 0.80 in patients with persistent AKI with a standard deviation of 0.06, using a two-sided test with an α-risk (type I error) of 5% and a statistical power of 80%, we needed 26 patients to detect a 10% absolute difference in RI between patients with persistent AKI and those with transient AKI or without AKI. According to the requirements of a reliable logistic model, we assumed that three or four variables would be included in a prediction model: “nonrenal SOFA score”, “norepinephrine dose”, “fluid balance” or “comorbid diabetes”. Ten events per variable means we needed 30–40 events (patients with persistent AKI) [29]. Assuming a 40% incidence rate based on previous studies [10,16], 75–100 patients needed to be enrolled.

Logistic regression was performed to identify variables significantly associated with persistent AKI. The selected variables were “serum creatinine at inclusion”, “nonrenal SOFA score”, “lactate”, “fluid balance at 24 h” and “norepinephrine dose”, and they were selected according to their clinical relevance and statistical significance in the univariate analysis. A multivariable logistic regression analysis was then performed, and the variables were forced into the model (enter mode). The Hosmer Lemeshow test was used to check the goodness of fit of the logistic regression.

Discriminative power was evaluated in a receiver operating characteristic (ROC) curve analysis. The area under the receiver-operating characteristic curve (AUROC) was recorded, together with its 95% confidence interval (CI). AUROCs were compared in DeLong’s test [30]. We used the Youden index to determine the optimal cutoff for calculations of specificity, sensitivity, and positive and negative predictive performances.

All statistical analyses were performed using SPSS 21 (IBM, Armonk, NY, USA) and MedCalc version 16.8 (MedCalc Software, Ostend, Belgium), with a two-sided *p-*value <0.05 considered statistically significant.

## Result

### Population characteristics

**Figure 1.**
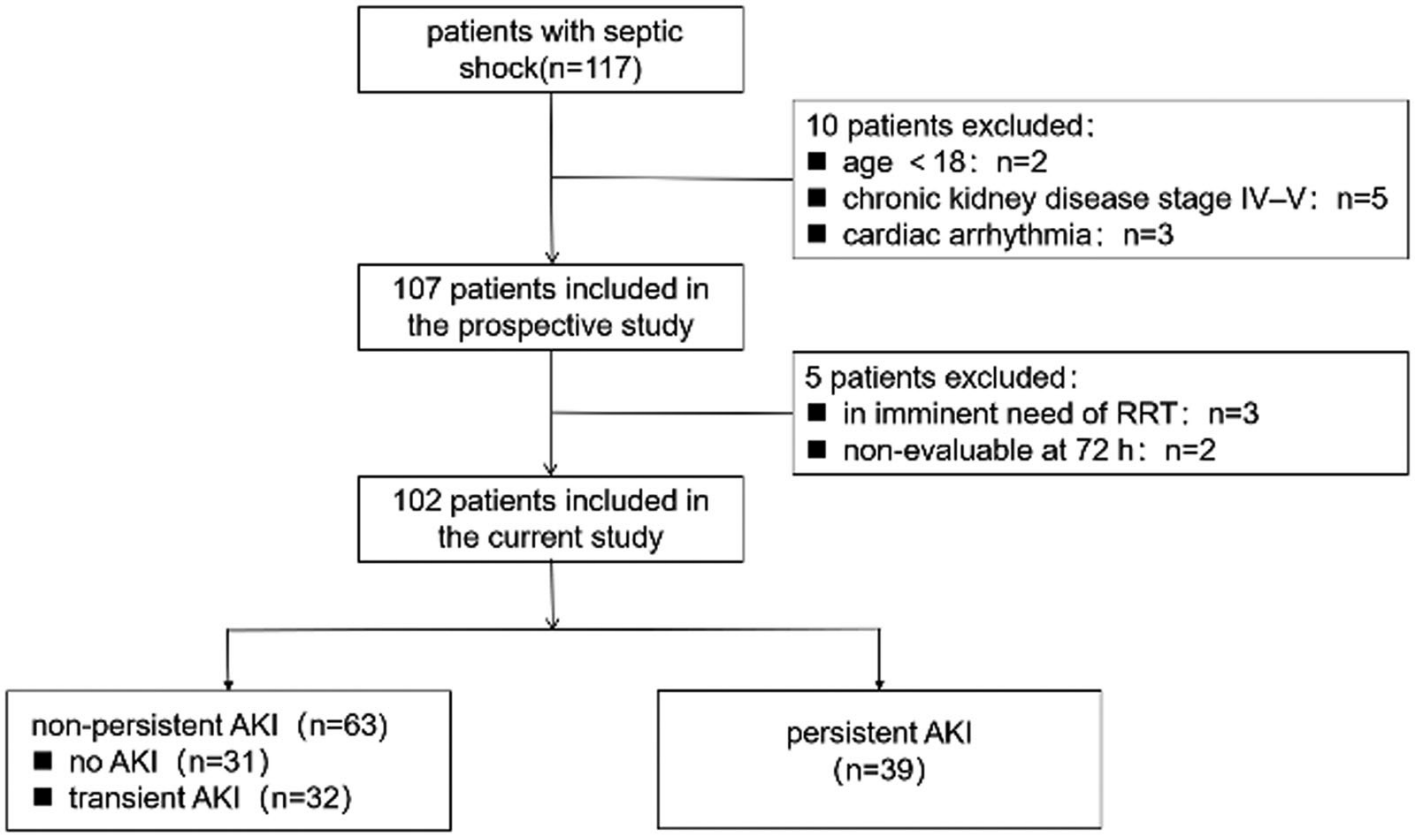
Flow chart. AKI: acute kidney injury.

We identified 102 patients who were enrolled for the final analysis (Figure 1). The characteristics of the 102 analyzed patients are shown in Table 1. The average age was 70 years, and approximately two-thirds were male. A total of 46 (45.1%) patients were admitted to the ICU after the operation, and 25 (24.5%) patients were from the emergency room, which is similar amount compared to that from the ward (28.4%). The presumed or confirmed sepsis sources were as follows: respiratory tract (40, 39.4%), abdominal (36, 35.3%), urinary tract (11, 10.8%), and other sites (15, 12.7%). The mean APACHE II and SOFA scores were 16.3 ± 4.8 and 8.5 ± 3.5, respectively. Nine patients received KRT in the ICU. The median ICU stay was 4.3 days, and the ICU mortality rate was 26.0%.

**Table 1.** Patient characteristics.

	All patients (*n* = 102)	no P-AKI (*n* = 63)	P-AKI (*n* = 39)	*p* Value
Demographic data				
Sex, male	69 (67.6)	42 (66.7)	27 (69.2)	0.788
Age, yr	70 ± 15	70 ± 14	71 ± 17	0.826
Hypertension	57 (55.9)	33 (52.4)	24 (61.5)	0.365
Mode for ICU admission				0.054
Emergency room	25 (24.5)	17 (27.0)	8 (20.5)	
Operating room	46 (45.1)	32 (50.8)	14 (35.9)	
Ward	29 (28.4)	14 (22.2)	15 (38.5)	
Transferred from another center	2 (2.0)	0 (0.0)	2 (5.1)	
Primary source of sepsis				0.095
Pulmonary	40 (39.4)	22 (34.9)	18 (46.2)	
Intra-abdominal	36 (35.3)	28 (44.4)	8 (20.5)	
Urinary	11 (10.8)	5 (7.9)	6 (15.4)	
Other	15 (12.7)	8 (12.7)	7 (17.9)	
Time from vasopressor initiation to inclusion, h	3.0 [2.0–5.0]	3.0 [2.0–5.0]	3.0 [1.0–5.0]	0.500
Clinical characteristics at inclusion				
Heart rate, beats/min	110 ± 22	108 ± 23	115 ± 19	0.170
MAP, mmHg	79 ± 15	80 ± 14	77 ± 15	0.269
Hemoglobin, g/L	112 ± 28	113 ± 30	109 ± 26	0.508
Platelet, ×10^9^ /L	174 ± 103	190 ± 103	147 ± 99	0.039
Total bilirubin, μmol/L	15.5 [9.7–24.1]	12.8 [9.2–20.2]	19.5 [12.2–41.1]	0.004
PaO_2_/FiO_2_, mmHg	225 ± 100	233 ± 97	212 ± 104	0.301
PaCO_2_,mmHg	40 ± 14	40 ± 13	39 ± 16	0.599
Lactate, mmol/L	2.3 [1.7–3.6]	1.9 [1.4–3.0]	2.3 [3.4–6.1]	<0.001
Procalcitonin, ng/mL	6.8 [1.5–16.9]	5.4 [1.0–12.1]	9.6 [1.8–34.0]	0.030
CVP, mmHg	9.9 ± 4.6	9.2 ± 4.7	11.0 ± 4.3	0.047
APACHEII score	16.3 ± 4.8	16.1 ± 4.6	16.7 ± 5.2	0.586
SOFA score	8.5 ± 3.5	6.9 ± 2.4	11.0 ± 3.6	<0.001
nonrenal SOFA score	7.8 ± 3.3	6.6 ± 2.4	9.7 ± 3.5	<0.001
Kidney function				
Baseline sCr, μmol/L	72.7 ± 16.1	70.6 ± 17.4	76.1 ± 13.3	0.096
Based back-calculation	23 (22.5)	12 (19.0)	11 (28.2)	0.282
Serum creatinine, µmol/L	119.5 ± 72.5	91.5 ± 34.3	160.6 ± 92.5	<0.001
Organ support and treatment at inclusion				
Norepinephrine dose, µg/kg/min	0.25 [0.10–0.50]	0.20 [0.10–0.35]	0.48 [0.14–0.90]	0.006
Mechanical ventilation	92 (90.2)	57 (90.5)	35 (89.7)	1.000
Time from inclusion to hemodynamic stabilization, h	1.0 [0.0–3.0]	1.0 [0.0–2.0]	3.0 [1.0–5.0]	<0.001
Persistent AKI risk factors				
Diabetes mellitus	30 (29.4)	14 (22.2)	16 (41.0)	0.043
Chronic kidney disease	18 (17.6)	11 (17.5)	7 (17.9)	0.950
Fluid balance at 24 h,ml/kg	35.2 ± 33.7	22.8 ± 22.6	45.6 ± 44.8	0.029
Use of diuretics at 24 h	7 (6.9)	5 (7.9)	2 (5.1)	0.705
Nephrotoxic agents	20 (19.6)	9 (14.3)	11 (28.2)	0.085
Renal RI	0.67 ± 0.06	0.66 ± 0.05	0.70 ± 0.05	0.001
KRT during hospitalization	9 (8.8)	0 (0)	9 (23.1)	<0.001
Length of ICU stay, days	4.3 [2.7–7.3]	4.0 [2.2–7.8]	4.3 [2.7–7.2]	0.608
Death in the ICU	26 (26.0)	8 (25.5)	18 (46.2)	<0.001

Values represent means ± SDs, n (%), or medians [IQR].

IQR: interquartile range; P-AKI: persistent acute kidney injury; MAP: mean arterial pressure; PaO_2_/FiO_2_: arterial oxygen partial pressure to fractional inspired oxygen; CVP: central venous pressure; APACHE II: Acute Physiology and Chronic Health Evaluation II; SOFA: Sequential Organ Failure Assessment; sCr: serum creatinine; RI: resistive index; KRT: kidney replacement therapy.

According to our definitions, the P-AKI group was comprised of 39 patients who developed persistent AKI, the no P-AKI group was comprised of 31 patients who had transient AKI and there were 32 patients who did not develop AKI. There were no differences in age or sex between the two groups. The P-AKI group had a higher proportion of patients with diabetes mellitus (41.0% vs. 22.2%, *p* = 0.043) but did not have a higher proportion of patients with hypertension and chronic kidney disease. The mode of ICU admission, primary source of sepsis and time from vasopressor initiation to inclusion were similar between the groups. Some physiological indexes and laboratory measurements, such as CVP, sCr at inclusion, platelets, total bilirubin, lactate concentration, and procalcitonin, were significantly different between the groups (Table 1). The patients with persistent AKI had higher SOFA scores (11.0 ± 3.6 vs. 6.9 ± 2.4; *p* < 0.001) and nonrenal SOFA scores (9.7 ± 3.5 vs. 6.6 ± 2.4; *p* < 0.001) at inclusion. In the patients with persistent AKI, a higher norepinephrine dose (0.48 [0.14–0.90] vs. 0.20 [0.10–0.35]); *p* = 0.006) and more fluid (45.6 ± 44.8 vs. 22.8 ± 22.6); *p* = 0.029) were needed; KRT was initiated in 23.1% of the patients; and their ICU mortality was significantly increased (46.2% vs. 25.5%; *p* < 0.001). However, their ICU stay duration did not differ (4.3 [2.7–7.2] vs. 4.0 [2.2–7.8]; *p* = 0.608).

### Significant clinical indicators associated with persistent AKI

According to the clinical relevance and statistical significance of the variables in the univariate analysis, “serum creatinine at inclusion”, “nonrenal SOFA score”, “lactate” and “norepinephrine dose” were forced into a model for the prediction of persistent AKI. Based on logistic regression analysis, “serum creatinine at inclusion” and “nonrenal SOFA score” had a significant association with persistent AKI (Table 2). When “lactate” and “fluid balance at 24 h” were forced to combine with “serum creatinine at inclusion” and “nonrenal SOFA score”, they were not independently associated with persistent AKI, and neither were “fluid balance at 24 h” and “norepinephrine dose”. Therefore, the final model included serum creatinine at inclusion (OR, 1.03; 95% CI 1.01–1.04) and nonrenal SOFA score (OR, 1.48; 95% CI 1.21–1.81).

**Table 2.** Logistic regression models for the prediction of persistent AKI.

Variable included	Univariate analysis		Multivariable analysis		Clinical Model 2		Clinical Model 3		Clinical model 4	
	OR (95% CI)	*p* Value	Clinical Model 1		OR (95% CI)	*p* Value	OR (95% CI)	*p* Value	OR (95% CI)	*p* Value
			OR (95% CI)	*P* value						
sCr at inclusion	1.03 (1.01–1.04)	<0.001	1.02 (1.01–1.04)	0.002	1.03 (1.01–1.04)	<0.001	1.03 (1.01–1.04)	0.001	1.03 (1.01–1.04)	<0.001
Nonrenal SOFA score	1.44 (1.22–1.69)	<0.001	1.41 (1.09–1.83)	0.009	1.47 (1.18–1.82)	0.001	1.40 (1.10–1.80)	0.007	1.48 (1.21–1.81)	<0.001
Lactate	1.72 (1.30–2.27)	<0.001	1.41 (0.96–2.08)	0.079	1.44 (0.99–2.10)	0.057	–	–	–	–
CVP	1.10 (0.99–1.20)	0.053	–	–	–	–	–	–	–	–
Fluid balance at 24 h	1.012 (1.00–1.03)	0.021	–	–	1.00 (0.98–1.03)	0.686	1.01 (0.99–1.03)	0.443	–	–
Norepinephrine dose	12.19 (2.54–58.61)	0.002	2.34 (0.26–20.78)	0.446	–	–	3.67 (0.42–30.44)	0.229	–	–

AKI: acute kidney injury; sCr: serum creatinine; SOFA: sequential organ failure assessment; CVP: central venous pressure; OR: odds ratio; CI: confidence interval.

Hosmer Lemeshow goodness of fit for clinical Model 1: χ^2^ = 9.245, df = 8, *p* = 0.322; Model 2: χ^2^ = 12.57, df = 8, *p* = 0.128; Model 3: χ^2^ = 14.354, df = 8, *p* = 0.073; Model 4: χ^2^ = 9.262; df = 8; *p* = 0.321.

### Performance of renal resistive index, clinical indicators and their combination for predicting persistent AKI

**Figure 2.**
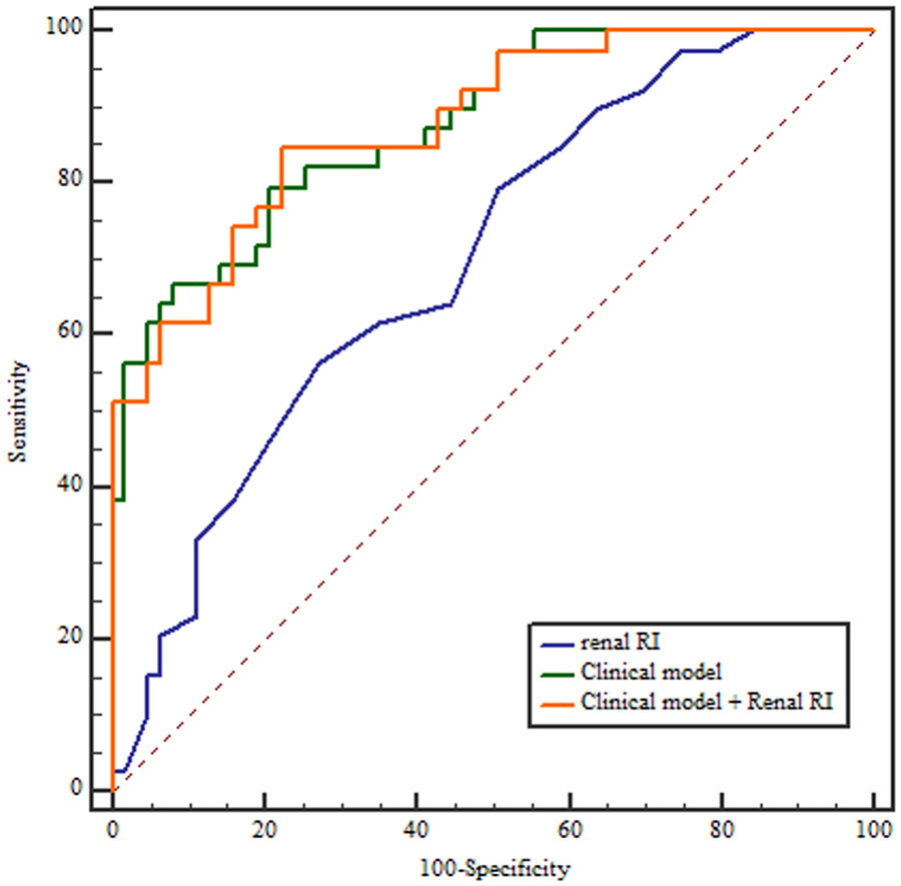
ROC curve reflecting performance of renal resistive index, the clinical model and their combination for predicting persistent AKI Clinical model includes serum creatinine at inclusion and nonrenal Sequential Organ Failure Score (SOFA). RI: resistive index.

The renal RI was significantly higher in the patients with persistent AKI (0.70 ± 0.05 vs. 0.66 ± 0.05; *p* = 0.001). The performance of RI to predict persistent AKI was poor, with an AUROC (95% CI) of 0.699 (0.600–0.786). The sensitivity and specificity were 56.4% and 73.0%, respectively, for an optimal cutoff equal to 0.68. The clinical model combining serum creatinine at inclusion and the nonrenal SOFA score had a good performance for predicting persistent AKI, with an AUROC of 0.877 (0.797–0.933), which was significantly better than renal RI (z statistic = 3.076, *p* = 0.0021). When incorporated into these parameters, renal RI was not independently associated with persistent AKI (OR 1.09 per 0.01 unit; 95% CI 0.98–1.21, *p* = 0.116) and did not improve the performance of the clinical model for predicting persistent AKI (AUROC 0.877; 95% CI 0.797–0.934, *p* = 0.9749) (Figure 2 and Table 3).

**Table 3.** Performance of renal RI, the clinical model and their combination for predicting persistent AKI.

	Sensitivity (95% CI)	Specificity (95% CI)	PPV (95% CI)	NPV (95% CI)	AUROC (95% CI)	*p* Value
Renal RI	56.4 (39.6–72.2)	73.0 (60.3–83.4)	56.4 (39.6–72.2)	73.0 (60.3–83.4)	0.699 (0.600–0.786)	
Clinical model	79.5 (63.5–90.7)	79.4 (67.3–88.5)	70.5 (54.8–83.2)	86.2 (74.6–93.9)	0.877 (0.797–0.933)	0.0021^a^
Clinical model + Renal RI	84.6 (69.5–94.1)	57.1 (44.0–69.5)	55.0 (41.6–67.9)	85.7 (71.5–94.6)	0.877 (0.797–0.934)	0.9749^b^

The clinical model includes serum creatinine at inclusion and the nonrenal Sequential Organ Failure Score (SOFA). RI: renal index, AKI: acute kidney injury, AUROC: area under the receiver-operating characteristic curve, CI: confidence interval, PPV: positive predictive value, NPV: negative predictive value.

^a^Comparison between the AUROC of renal RI and the clinical model.

^b^Comparison between the AUROC of the clinical model and the clinical model + RRI.

The discriminative performance of renal RI was also assessed in the subgroup of patients presenting with AKI at inclusion, and it remained similarly limited in this subgroup, as well as in the patients whose baseline serum creatinine was calculated using different methods (Table 4). The percentage of patients aged 70 years or older was as high as 56.9% (58/102) in our study population. We also evaluated the predictive ability of renal RI in this subgroup, and the performance of RI was also not good, with an AUROC of 0.716 (95% CI 0.583–0.827) (Table 4).

**Table 4.** Performance of renal RI for predicting persistent AKI in different subgroups.

Subgroup	Sample	Cutoff value	Sensitivity (95% CI)	Specificity (95% CI)	PPV (95% CI)	NPV (95% CI)	AUROC (95% CI)
Presenting AKI at inclusion	58	0.65	76.7 (57.7–90.1)	60.7 (40.6–78.5)	67.6 (49.5–82.6)	70.8 (48.9–87.4)	0.717 (0.583–0.827)
Ag*e* ≥ 70 years	58	0.71	45.8 (25.6–67.2)	88.2 (72.5–96.7)	73.3 (44.9–92.2)	69.8 (53.9–82.8)	0.716 (0.583–0.827)
Baseline sCr based on the previous measurement	79	0.65	85.7 (67.3–96.0)	52.9 (38.5–67.1)	50.0 (32.5–64.8)	87.1 (70.2–96.4)	0.711 (0.599–0.808)
Baseline sCr based on the MDRD formula	23	0.69	54.6 (23.4–83.3)	100.0 (73.5–100.0)	100.0 (54.1–100.0)	70.6 (44.0–89.7)	0.682 (0.457–0.858)

RI: renal index; AKI: acute kidney injury; AUROC: area under the receiver operating characteristic curve; CI: confidence interval; PPV: positive predictive value; NPV: negative predictive value; sCr: serum creatinine: MDRD: Modification of Diet Renal Disease.

## Discussion

In this study, we evaluated renal RI and clinical indicators to predict persistent AKI in patients with septic shock. Renal RI was unable to predict persistent AKI and did not improve the prediction provided by the usual clinical variables. A combination of serum creatinine at inclusion and the nonrenal SOFA score showed good performance in prognosticating AKI.

Several studies suggested that the Doppler-based renal resistive index might help to separate transient from persistent AKI in critically ill patients [15,16]. A recent study including 100 patients with AKI showed a very good performance of RI in predicting short-term reversibility of AKI in patients in a medical ICU [31], and similar results were reported in a meta-analysis [17]. In our cohort, the results were different. Several points can explain the differences.

First, renal RI has been considered a valuable and repeatable marker of renal vascular resistance (RVR) and blood flow [13,32] and was proposed as a biomarker to monitor kidney perfusion in ICU patients [33]. However, experimental studies have shown that the association between Doppler-based RI and RVR was weak and that supraphysiological variations of resistance only translated into small RI variations clinically [34]. In addition, numerous confounding factors could influence RI values, including vascular compliance, MAP, heart rate, hypoxemia and hypercapnia [14,28,35–37]. In several studies, vascular compliance was a major determinant of RI [34,38,39]. Decreased vascular compliance may result not only from preexisting subclinical vascular stiffness (e.g. aging [40], hypertension [41], diabetes mellitus [42]) but also from acute changes in the renal central vasculature [28], renal interstitial pressures or intra-abdominal pressure [39,43]. The impact of intra-abdominal pressure on renal RI also comes from the possible contribution of the reduction in the renal perfusion due to the increase of intra-abdominal pressure [13,44]. Furthermore, the relationship between RI and RVR seemed to be linear only when the vascular compliance was normal, but this relationship progressively disappeared when the arterial stiffness increased [34,38]. Given the numerous factors influencing RI and its weak correlation to renal blood flow, the potential ability of RI to predict the reversibility of AKI seems questionable.

Second, AKI is a complex and heterogeneous clinical syndrome. Kidney damage varies according to the type of primary insult, secondary effects and mitigating responses and leads to distinct molecular, cellular and functional changes. Different subtypes of AKI with varying recovery patterns have been identified [45]. We focused on septic shock patients who were considered to be exposed to multiple kidney insults. The exact mechanism for sepsis-associated AKI is still under investigation. Macrovascular and microvascular dysfunction, immunological and autonomic dysregulation, and abnormal cellular response to injury have been proposed [11]. Along with these findings, previous studies suggested that a longer duration of AKI may reflect a more serious form of AKI rather than specific pathophysiological mechanisms (e.g. septic, nephrotoxic, inflammatory) [46,47]. Based on the above complex mechanisms and their interaction, renal RI, as a preliminary assessment of microvascular changes in the kidney, may lack the ability to predict kidney recovery. Saade et al. [48] performed a multicenter, prospective cohort study of 371 unselected ICU patients to evaluate the performance of RI to separate patients with transient AKI from patients with persistent AKI. Similar to our results, RI performed poorly in predicting persistent AKI, and it was not recommended that RI be used to assess short-term kidney prognosis in the ICU setting. In addition, in terms of assessing AKI severity, it was found that RI also may not be a good predictor of stage 3 AKI in the study conducted by Zhi et al. [49].

Our hypothesis was that clinical indicators could improve the ability of RI in predicting kidney recovery; however, RI performed poorly. Finally, the clinical model combining sCr at inclusion and SOFA score without a renal component was more effective for predicting short-term changes in kidney function. Considering the complexity of AKI, researchers have attempted to utilize different clinical variables combined with or without AKI biomarkers to construct clinical prediction models for AKI recovery. In a single center cohort of 57 patients with AKI, Dewitte [50] showed that when the nonrenal SOFA score, sCr at inclusion and fluid balance were selected to establish a clinical model, combinations of AKI markers in addition to this clinical model improved the discrimination of patients who would recover from AKI. Notably, this clinical model showed good predictive value for nonrecovery, with an AUROC of 0.87 (95% CI 0.78–0.9). Similarly, Jia et al. [51] found that the predictive accuracy of nonrecovery can be improved when [TIMP-2]*[IGFBP7] was combined with the clinical factors of AKI diagnosed by the urine output criteria, AKI stage 2–3 and nonrenal SOFA score. Additionally, Titeca-Beauport et al. [9] reported that a predictive model including sCr, nonrenal SOFA and two other clinical indicators had better discrimination in persistent AKI than [TIMP-2]*[IGFBP7] in the early phase of septic shock. In keeping with these findings, our study suggested that the nonrenal SOFA score and sCr at inclusion were independent predictors for persistent AKI and that their combination had good performance in prognosticating AKI.

The renal angina index (RAI), which is based on small changes in serum creatinine and patient conditions, is another remarkable predictive model. Analogous to myocardial angina, the concept of renal angina combines risk stratification and clinical signs to increase the pretest probability of AKI biomarkers [52]. In fact, RAI was suggested to be efficient in discriminating pediatric patients who will develop persistent AKI [53], and a similar score has been developed and validated in an adult setting and performed well in predicting the low risk of persistent AKI [54]. Additionally, incorporation of AKI biomarkers into RAI may potentially improve prediction [55]. However, several limitations, such as low event rate and unreported or poor calibration, make RAI controversial and difficult to be utilized clinically [56]. Because renal RI measurement is a noninvasive, bedside, rapid and repeatable tool, we still want to use it to assess kidney recovery, despite its poor performance in our study. Similar to the process of constructing RAI, future studies could aim to utilize RI to risk stratify patients or to preliminarily select the potential population of patients at high risk of persistent AKI, among whom AKI biomarkers or other predictive tools may be more economical and powerful.

Our study had a high proportion of elderly patients, and the subgroup analyses did not find a predictive advantage of RI. However, elderly patients might have a higher baseline SOFA score because of comorbidities, and Ferreira et al.[57] reported that compared with the admission SOFA score, the delta-SOFA score (differences between subsequent scores) was a better indicator of prognosis, especially in patients with chronic organ dysfunction. Therefore, dynamic changes in the SOFA score may be a better choice to develop a multivariable model instead of using a static value for this population. However, using a static value did not have an impact on the significance of our current prediction model because there was no difference in age between the two groups.

Our study has several limitations. First, our definition of persistent AKI differs from the recently suggested definition of the Acute Disease Quality Initiative group [7]. However, the design of our study was initiated before the publication of this definition. Second, eighteen patients no longer required norepinephrine when they arrived at the ICU because of previous infection control and fluid resuscitation in the emergency room or operating room. Sixteen of them did not develop persistent AKI, which may lead to bias in the statistical results. Third, we did not obtain RI prior to resuscitation because the change in RI before and after hemodynamic resuscitation may be a better indicator for predicting persistent AKI. However, the value of RI was unstable due to the impact of dynamic changes in MAP prior to hemodynamic resuscitation [58]. Fourth, we did not consider the effect of positive end-expiratory pressure and peak airway pressure on RI, which would lead to a high RI value [43,59]. Last, this was a single-center observational study focusing on septic shock patients, and the findings may not have sufficient external validation.

## Conclusion

In summary, we found that the Doppler-based renal resistive index performed poorly in predicting persistent AKI and did not improve the clinical prediction provided by a combination of serum creatinine at inclusion and nonrenal SOFA score in patients with septic shock. To take better advantage of renal RI, further studies are needed to risk stratify patients or deem RI as a preliminary screening tool.

## Data Availability

The datasets used and/or analyzed during the current study are available from the corresponding author on reasonable request.
